# Therapeutic silence of pleiotrophin by targeted delivery of siRNA and its effect on the inhibition of tumor growth and metastasis

**DOI:** 10.1371/journal.pone.0177964

**Published:** 2017-05-31

**Authors:** Lisha Zha, Lichun He, Weidong Xie, Jin Cheng, Tong Li, Mona O. Mohsen, Fan Lei, Federico Storni, Martin Bachmann, Hongquan Chen, Yaou Zhang

**Affiliations:** 1 School of Animal Science and Technology, Anhui Agricultural University, Hefei, Anhui, P.R. China; 2 Key Lab in Healthy Science and Technology, Division of Life Science, Graduate School at Shenzhen, Tsinghua University, Shenzhen, P.R. China; 3 The Jenner Institute, Oxford University, Oxford, United Kingdom; 4 Laboratory of Pharmaceutical Science, School of Life Science, School of Medicine, Tsinghua University, Beijing, China; 5 Department of Immunology, Inselspital, University of Bern, Salihaus 2, Bern, Switzerland; 6 Open FIESTA Center, Tsinghua University, Shenzhen, P.R. China; Rutgers University, UNITED STATES

## Abstract

Pleiotrophin (PTN) is a secreted cytokine that is expressed in various cancer cell lines and human tumor such as colon cancer, lung cancer, gastric cancer and melanoma. It plays significant roles in angiogenesis, metastasis, differentiation and cell growth. The expression of PTN in the adult is limited to the hippocampus in an activity-dependent manner, making it a very attractive target for cancer therapy. RNA interference (RNAi) offers great potential as a new powerful therapeutic strategy based on its highly specific and efficient silencing of a target gene. However, efficient delivery of small interfering RNA (siRNA) in vivo remains a significant hurdle for its successful therapeutic application. In this study, we first identified, on a cell-based experiment, applying a 1:1 mixture of two PTN specific siRNA engenders a higher silencing efficiency on both mRNA and protein level than using any of them discretely at the same dose. As a consequence, slower melanoma cells growth was also observed for using two specific siRNA combinatorially. To establish a robust way for siRNA delivery *in vivo* and further investigate how silence of PTN affects tumor growth, we tested three different methods to deliver siRNA *in vivo*: first non-targeted *in-vivo* delivery of siRNA *via* jetPEI; second lung targeted delivery of siRNA *via* microbubble coated jetPEI; third tumor cell targeted delivery of siRNA *via* transferrin-polyethylenimine (Tf-PEI). As a result, we found that all three *in-vivo* siRNAs delivery methods led to an evident inhibition of melanoma growth in non-immune deficiency C57BL/6 mice without a measureable change of ALT and AST activities. Both targeted delivery methods showed more significant curative effect than jetPEI. The lung targeted delivery by microbubble coated jetPEI revealed a comparable therapeutic effect with Tf-PEI, indicating its potential application for target delivery of siRNA *in vivo*.

## Introduction

Melanoma is the deadliest skin cancer, which is an increasing worldwide health problem because of its highly aggressive and drug-resistant nature. The 5-year survival rate for melanoma patients with advanced disease is <15%[1]. A major biological characteristic of metastatic melanomas is their ability to survive in a growth factor lacking environments. They used the autocrine signalling to secret cytokines and growth factor for self-stimulation[2]. In addition, recent research also suggests that melanoma may activate normally dormant embryonic stem cell pathways to contribute to its tumorigenicity[3]. Pleiotrophin (PTN) might be one of the cytokines that melanomas secreted for autocrine signalling[4]. Originally, PTN is highly expressed in the central and peripheral nervous system during embryonic and early postnatal development[5]. It plays multiple roles in neuronal development, hepatic regeneration, bone repair, skeletal remodeling and other physiological processes[6–9]. In adult, the expression of PTN is strongly down-regulated in the differentially cells, persisting only in an activity-dependent manner in the hippocampus[10]. However, in many different types of cancer, such as melanomas, breast cancer, gliomas, neuroblastomas, prostate cancer, leukemias, meningiomas, choriocarcinomas and lung cancer[11–14], PTN is highly expressed and identified to play a critical role in angiogenesis, tumor cell proliferation and metastasis[11,15]. Thus PTN becomes an attractive target for cancer gene therapy.

RNA interference (RNAi) has been widely used for *in vitro* gene silencing and offers great potential as a novel therapeutic strategy in vivo[16]. However, the systemic application of siRNA still faces serious challenges including the stability problem of siRNA in the bloodstream, target delivery of siRNA to specific tissues as well as cells, and its transportation across the cell membrane. Chemical modification of various molecule postions of siRNA have been reported to extend its half-lives in plasma and cell culture, such as introducing O-methyl (2'-O-Me), fluoro (2'-F) group or 2'-deoxy group[17]. Conventional siRNA delivery approaches including systemically application of liposome, jetPEI and Tf-PEI. Polyethylenimine (PEI) is a cationic polymer in linear (jetPEI) or branched form. Low molecularweight and linear PEI is used as a carrier of siRNA to facilitate cellular internalization with minimal toxicity[18]. Transferrin-polyethylenimine (Tf-PEI) is the conjugated polyethylenimine with transferrin protein through covalent bond. This tranferrin–PEI takes the advantage of highly expressed transferrin receptors (TfR) on many cancer cells[19]. TfR is a membrane protein intrinsically bound to the iron transport protein transferrin that consequently leads to the targeted delivery of siRNA to cancer cells by Tf-PEI conjugation. However the requirement of endocytosis for jetPEI and Tf-PEI to get into the cytosol could hinder their efficiency. One novel approach to the administration of siRNA is ultrasound (US)-enhanced delivery by microbubbles. The ultrasound generates inertial cavitation improving the permeability of cell membranes and enabling extracellular molecules transport directly into the cytosol[20]. In addition, large microbubbles with diameter more than 10 μm will collapse and release its contents into lung capillary bed leading to the lung targeted release[21]. Animal studies have shown that these microbubbles don't affact hemodynamic and flow at similar velocity as red blood cells if they are injected into a peripheral vein[22,23] Thus a combination of microbubbles with conventional jetPEI for gene silencing *in vivo* tends to be highly interested.

So far, two studies exploring anti-PTN antisense RNA[14] and ribozyme targeting[4] to establish PTN as an attractive target for the therapy of melanoma. Inhibtion of PTN expression led to the reduced subcutaneous tumor growth and metastatis to lung of 1205Lu cells in nude mice[4]. In the present study, we first determined that PTN is highly produced in the melanoma cells and then sought to (1) investigate the silencing efficient of the cocktail of two PTN specific RNA (2) study the effect of the PTN expression level on melanoma cell proliferation (3) most importantly test clinically more relevant systemic administrations of siRNA *in vivo*. We found that the microbubble coated jetPEI-siRNA led to more significant inhibition of metastatic tumor growth over jetPEI-siRNA complex. It showed a comparable therapeutic effect with the cancer cell targeted Tf-PEI-siRNA complex, revealing a potential lung-targeted delivery way of siRNA.

## Material and method

### Ethics statement

Six week old male C57BL/6 nude mice were purchased from Beijing Vital River Laboratory Animal Technology Co., Ltd (Beijing, China). The animals were kept in an environmentally controlled breeding room (temperature, 20±2°C; humidity, 60%±5%; dark/light cycle, 12 h). The mice were fed with standard laboratory chow and water ad libitum. The experiment was performed in strict accordance with the recommendations in the Guide for the Care and Use of Laboratory Animals of the Institutional Animal Care and Use Committee of Tsinghua University, Beijing, China. The protocol was approved by the Animal Welfare and Ethics Committee of Tsinghua University. All animals were fasted from 09:00h to 15:00 h before the experiments. For the animal sacrifice, two-Step euthanasia (CO_2_ narcosis followed by a physical neck break) was used. Cervical dislocation was used to kill rodents after they have been sedated with CO_2_.

### siRNA design and cell culture

The target sequences of PTN-siRNA were: S1,Sense 5’-GCG GAG UCA AAG AAG AAG A dTdT-3’; Antisense 5’-UCU UCU UCU UUG ACU CCG C dTdT-3’; S2,Sense5’-GAC UCA GAG AUG UAA GAU C dTdT-3’; Antisense 5’-GAU CUU ACA UCU CUG AGU C dTdT-3’. A small RNA with random sequence was used as negative control (NC). All the siRNA and random sequences were synthesized by Shanghai GenePharma Co. (Shanghai, China) with 2'-deoxy modification. B16-F10 (ATCC® CRL-6475™) cells were cultured in Gibco™ RPMI 1640 Medium supplemented with 10% fetal bovine serum at 37°C in a humidified 5% CO_2_ incubator. The B16-F10 melanoma cells cultured on 6 well plates were transfected with siRNA or random sequence at a concentration of 100 pmol per well using Lipofectaime 2000 (Invitrogen, USA) according to the manufacturer’s instruction. 36 hours later, the cells were harvested for total RNA isolation and protein isolation.

### Quantitative real time RT-PCR and western blotting

48 hours After transfection, B16-F10 cells were washed by PBS buffer and RNA was isolated according the standard manufacturer’s protocol with TRIzol reagent (Invitrogen, USA)[24]. cDNA were synthesized by TaKaRa’s One Step RNA PCR kit (TaKaRa Dalian, China) with random primers and quantitative reverse transcription polymerase chain reaction (RT-PCR) of cDNA was performed to evaluate the silence efficiency of the siRNA. The expression of PTN was normalized to GAPDH expression. For protein immune blotting, B16-F10 cells were lysed with an ice-cold cell lysis buffer (50mM Tris-HCl pH8.0, 4M Urea and 1% Triton X-100), containing protease and phosphatase inhibitors (Roche, 04693132001), 48 hours after the transfection of PTN-siRNA. Equal amount of cell lysates were loaded on the SDS-PAGE and then transferred onto the nictrocellulose (NC) membrane. The NC membrane were then blocked with 5% milk and incubated with the primary goat anti-PTN polyclonal antibody (Oncogene). The secondary horseradish peroxidase-coupled anti-goat antibody (Santa Cruz) was applied and the protein bands were detected with ECL blotting detection reagents (KPL 547100). The intensity of the gel bands of both RT-PCR and western blotting were quantified later and the signal to noise level was used to calculate the error[25]. Western blotting of the lung lysate was also performed in the same way, except the lung tissue was homogenized by grinding first.

### Cell proliferation and viability assay

Trypan blue assay was performed to determine the cell proliferation rate. 5,000 B16-F10 cells were seeded in each well of 96-well plate. 6 hours after the cells attached on the plate, PTN siRNA were transfected according to the standard manufacture protocol with Lipofectaime 2000 (Invitrogen, USA)[26]. The serum free medium was replaced after 12h. Cells were detached by using trypsin-EDTA and resuspended in PBS buffer first and then mixed with 0.4% trypan blue solution after 24h, 48h and 72h incubation. The viable cells was quantified using Nexcelom Cellometer Mini Cell Counter. Each group was measured in triplicate. Cell numbers were expressed as mean ± standard deviation (SD). To evaluate the effect of recombinant PTN on cell proliferation, B16-F10 cells was seeded in 24-well plate with and without pre-incubation with 120ng/ml receptor protein tyrosine phosphatase beta/zeta (RPTPβ/ζ) specific antibody (anti-RPTPZ, ThermoFisher Scientific) for 6 hours. Then all the cells were treated with 100 ng/ml PTN. Cells proliferation and viability were then measured by the same trypan blue assay.

### Preparation of microbubbles and polyplexes

60% glucose(w/v) in water were pre-saturated by CO_2_ and then the sonication was applied to agitate the microbubble[27]. The sonicator tip was placed approximately 0.5–1 cm beneath the surface of the 60% glucose solution in a 50 mL falcon tube. Ultrasonic energy was delivered at a power of 60% for 10 s and the sonicator tip was moved up to the surface of the liquid to perform the surface agitation for 20 s. The whole process was repeated several times till a dense white, opaque coloration appeared. The size of the microbubble before and after filtering through the grade 595 1/2 filter paper (GE Whatman) was then determined by microscope attached with a charge-coupled device (CCD) camera. 40 μg of PTN-siRNA or random sequence was mixed with in vivo-jetPEI (Polyplus) at an N/P ratio of 6 in 60% glucose solution for tail vein injection. For microbubble-jetPEI-siRNA complex, additional sonication was applied as mentioned above and then used for tail veil injection. Tf–PEI Kit was purchased in Bender Medsystems. 40 μg of siRNA or random sequence was mixed with Tf-PEI (Bendermed Systems) at an N/P ratio of 5.2 in 1 × HBS solution for tail vein injection as recommended by the manufacturer.

### Mice and tumor growth assays

For B16 lung metastasis model, mice were injected with 0.4 × 10^6^ B16-F10 tumor cells intravenously. Three days later, after the tumor implantation, mice were randomly divided into 7 groups and treated systemically with saline or 40 μg of 2'-deoxy modification siRNA or random sequence (~2 mg/kg) in complex with TF-PEI, jetPEI and microbubble-jetPEI respectively. Intravenous injections of siRNA were repeated every third day for 2 weeks. Lungs were harvested, fixed and the number of B16 tumor colonies was manually counted. The aspartate transaminase (AST) and alanine transaminase (ALT) activities were measured in each mouse blood sample with the AST/ALT kit from Biosino Bio-Technology and Science Incorporation. According to the instruction, the normal AST serum level is between 0–100 UI/L and the normal ALT serum level is between 0–50 UI/L. All *in vivo* experiments are reported according to the guideline for describing laboratory-based animal research (**S1 Checklist)**.

### Data analysis

Quantitative data are achieved by counting the numbers of the metastatic melanoma colonies in lung in 5 different classes: > 2 mm, 1.5–2 mm, 1–1.5 mm, 0.5–1.0 mm, < 0.5 mm. The number of metastatic melanoma colonies were summed up by using different weight coefficient for each group. The weight coefficient is proportional to the square of the colonies diameters since the height of the lung tissue between two glasses was the same. Statistical significance was analysed by Student's t-test. P-values less than 0.05 were considered as statistically significant.

## Results

### Recombinantly expressed PTN stimulates B16-F10 cell proliferation through the RPTPβ/ζ receptor

**Fig 1:**
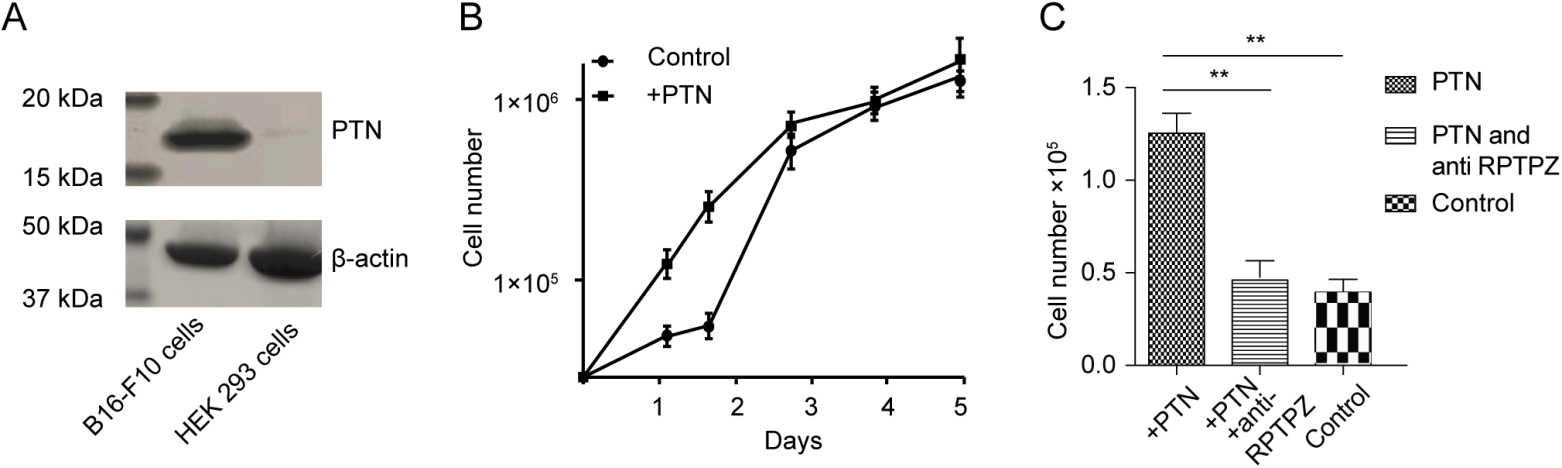
PTN expression level and its stimulation effect on the proliferation of B16-F10 melanoma cells. (A). Western-blot analysis of the expression level of PTN in B16-F10 melanoma cells and HEK293 cells. β-actin was detected as a load control. (B). Effects of recombinant PTN on B16-F10 melanoma cell proliferation. Each point represented the mean ± standard deviation of 3 replicates. PTN stimulated B16-F10 melanoma cells proliferation before the cells reached full confluency at a concentration of 100 ng/ml (P < 0.01 compared with control without adding the extracellular PTN). (C). Cells numbers of B16-F10 melanoma 24 hours after different treatments were counted and compared with the control group. Each bar represented the mean ± standard deviation of 3 replicates. Asterisks indicate statistically significant differences between samples (** p<0.01).

As mentioned in the introduction, PTN promotes the invasion and metastasis in many different tumor types including melanoma. To explore the expression level of PTN in melanoma tumor cell lines (B16-F10), western blotting of B16-F10 cells lysate was performed and compared to the PTN expression level in HEK293 cell line. Highly expressed PTN was observed in B16-F10 cells (Fig 1A), but not in HEK293 cell line. β-actin is used as negative control. Then we investigated whether recombinant expressed PTN regulates the proliferation of the B16-F10 cells in vitro. A final concentration of 100 ng/ml PTN was added into the cell culture medium to observe the cell growth rate. The growth curve revealed the addition of the recombinant PTN enhanced B16-F10 cells proliferation (Fig 1B), suggesting B16-F10 cell growth was still responsive to PTN stimulation and could employ it for autocrine signaling. However, if B16-F10 cell were pre-incubated with 120 ng/ml RPTPβ/ζ specific antibody first, its proliferation rate was significantly reduced upon stimulation by PTN (Fig 1C), revealing that PTN promotes B16-F10 cells proliferation through the RPTPβ/ζ receptor.

### Cocktail of two PTN siRNA leads to a higher silence efficiency

**Fig 2:**
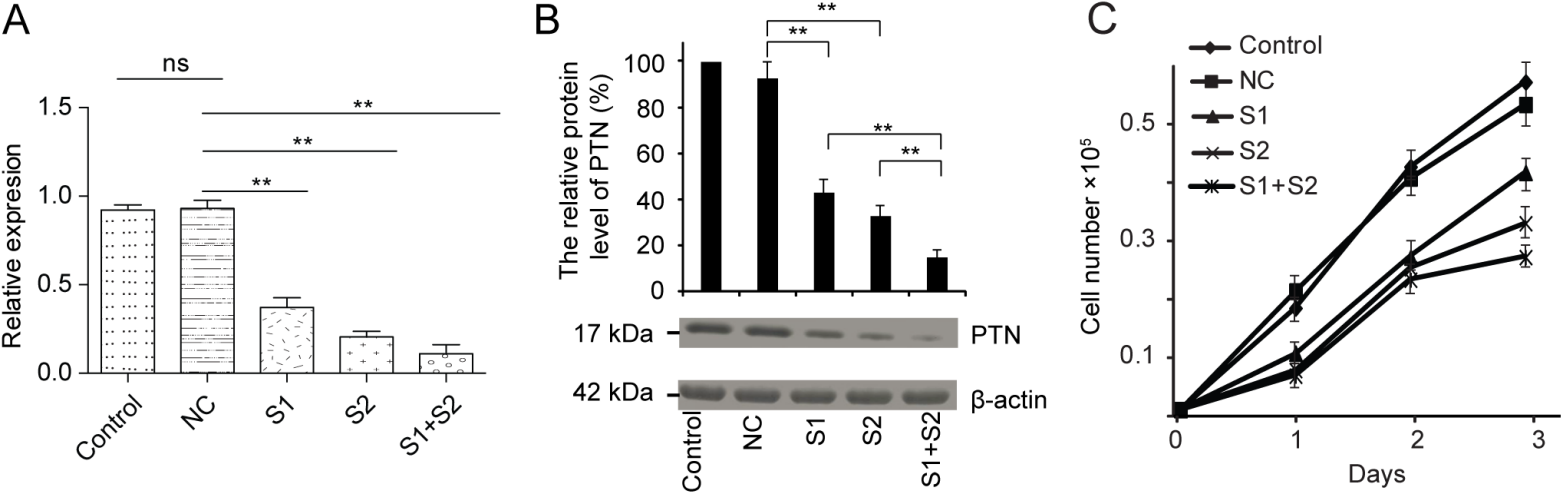
The silence efficiency of PTN siRNA measured by quantitative RT-PCR and western blotting. **The PTN expression level showed a direct correlation with the proliferation rate of B16-F10 melanoma cells.** (A). Quantitative RT-PCR analysis of the knock-down efficiency of siRNA 1, siRNA 2 and the mixture of them at the same dose. GAPDH was used as a control. (B). Western blotting analysis of the knock-down effect of siRNA 1, siRNA 2 and the mixture of them at the same dose. β-actin was used as a control. The intensity of the control sample without transfection of any siRNA was set as 100%. Asterisks indicate statistically significant differences between samples (** p<0.01 and ns for no significant difference). (C). Silence of the PTN by siRNA reduced the proliferation rate of B16-F10 melanoma cells in a manner directly related the reduced level of PTN. Each point represents the mean ± standard deviation of 3 replicates.

Two PTN specific siRNA oligonucleotides were designed to down regulate its expression in melanoma cells. Both sequences target the region upstream of residue 157 so that the expression of both isoforms of PTN would be antagonized. A standard 2'-deoxy modification was introduced into these two sequences and tested in vitro for the activity. The B16-F10 cells were transiently transfected with either a single siRNA or a combination of two siRNA at the same dose. The PTN expression was analyzed by quantitative RT-PCR and western blotting 48 h following the transfection. As shown in Fig 2A, the mRNA level of PTN protein was significantly decreased up to 80% by 100 pmol of siRNA. Compared to a single siRNA, the combination of the same amount of two siRNAs was highly effective and led to a more significant silence of PTN. The results were also confirmed by western blot analysis of PTN expression level (Fig 2B).

Next, we determined the effect of down regulation of PTN by siRNA on cell viability and proliferative ability using an trypan blue assay (Fig 2C). The numbers of viable B16-F10 cell treated with single or two siRNAs were lower than that of the random sequence treated cells. Moreover, the proliferative ability showed a direct correlation with the level of PTN. The dual-siRNA-treated cells has a stronger effect on the inhibition of cell viability, proliferative capacity. These cell culture studies indicate that more efficient inhibition of B16-F10 cell growth can be achieved by using two siRNAs combinatorially (siRNAc).

### Cancer cell targeted delivery of siRNAc by TfPEI and lung targeted delivery of siRNAc by microbubble-jetPEI significantly inhibit the metastasis of melanoma in lung

**Fig 3:**
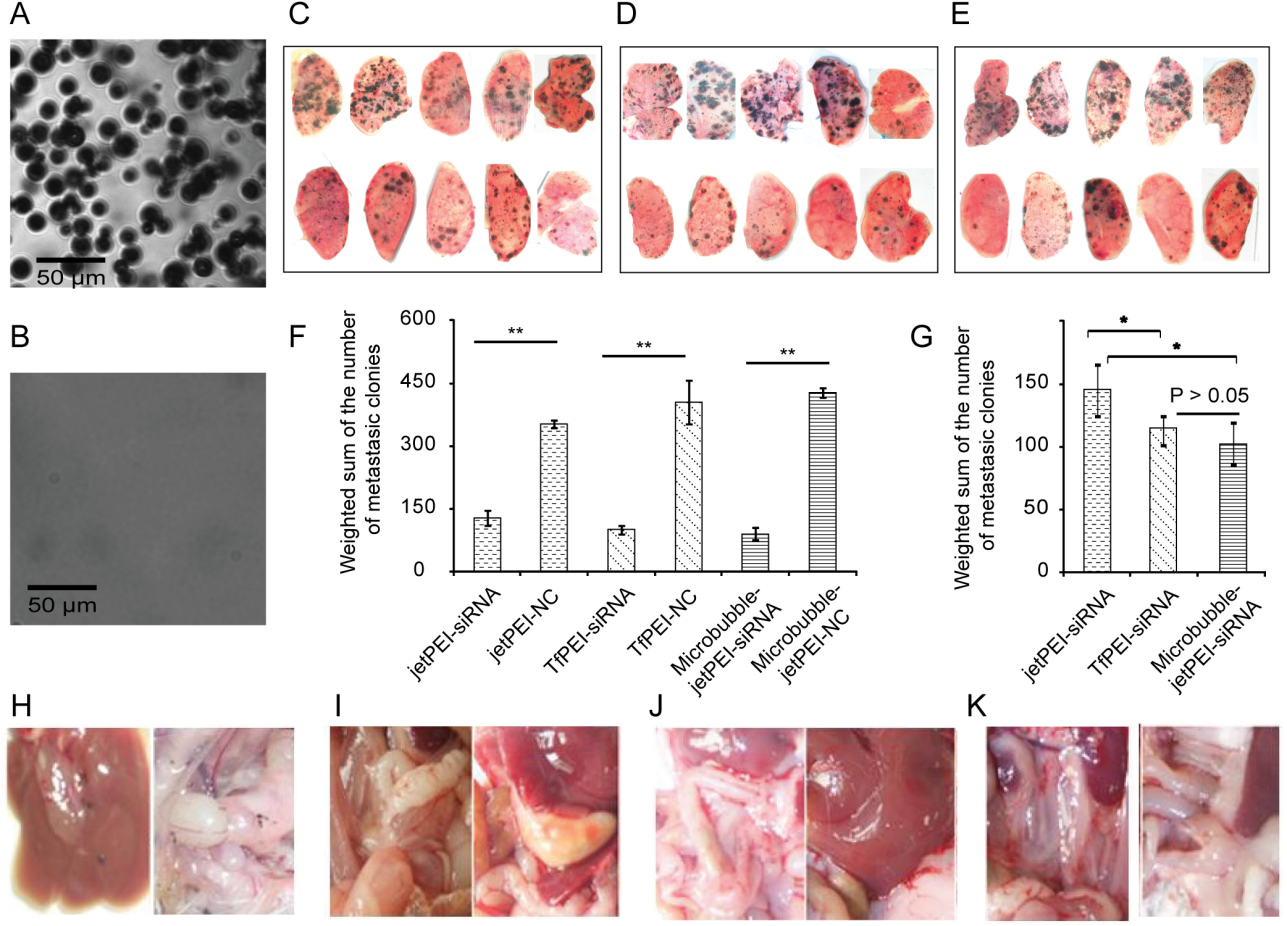
All three different systemic treatments by PTN siRNAc inhibited the tumor growth. (A). The microscopic image of the 60% glucose microbubble with 25x magnification. (B). The microscopic image of the 60% glucose microbubble filtered through the grade 595 1/2 filter paper with 25x magnification. Scale bar is 50 μm for both images. (C-E). Therapeutic effect of the systemic treatment of PTN siRNAc by using jetPEI, TfPEI and microbubble-jetPEI. (C). Lung of the mice treated with jetPEI-random sequence (Top) and jetPEI-siRNAc (Bottom). (D). Lung of the mice treated with TfPEI-random sequence (Top) and TfPEI-siRNAc (Bottom). (E). Lung of the mice treated with microbubble-jetPEI random sequence (Top) and microbubble-jetPEI siRNAc (Bottom). (F-G). The weighted sum of the metastatic colony numbers was plotted in bar chart as mean± standard deviation of 5 mice in each group. (G). The comparison of the siRNAc treatment group and the NC treatment group with the same delivery method. (F). The comparison between different siRNAc delivery methods. Asterisks indicate statistically significant differences between samples (** p<0.01 and * p<0.05) (H-K). Melanoma metastasis in liver and gastrointestinal tract. (H). The mice treated with random sequence showed a tumor metastasis in the gastrointestinal tract and liver. (I-K). The mice treated with siRNAc by systemic using of all three *in-vivo* transfection agent: (I). jetPEI-siRNAc, (J). TfPEI-siRNAc, (K). microbubble-jetPEI siRNAc revealed no tumor metastasis in the gastrointestinal tract and liver.

For the next step, we investigated the therapeutic effect of the combination siRNA treatment *in vivo*. In a pilot assay before performing the actual experiments, we observed that 14 days after the injection of the melanoma cell intravenously, the mice started to show a symptom. After killing the mice, the metastatic melanoma colonies could be visually observed in lung. So we decided to apply a treatment protocol with repeated intravenous injections of combination siRNA every third day for 2 weeks. The lung metastasis of melanoma is also quite common in clinic cases, because the lung has the smallest and most numerous blood vessels with diameter ranging from 4–9 μm, with a mean of 5 μm diameter[28]. This narrow pulmonary capillaries inspired us to coat the jetPEI-siRNA complex with microbubbles which could crash in the pulmonary capillaries to directional release of the jetPEI-siRNA complex in the lung. We applied the sonication to 60% glucose(w/v) solution pre-saturated by CO_2_ to agitate the microbubble and then measured the size of the microbubbles in light microscope images. According to the manuals of the microscope, one micrometer corresponds to 3.75 pixels in the final image by using a 25× objective. Fig 3A revealed the average diameter of the microbubbles we prepared was around 12±4 μm. Then we filtered the microbubbles through the grade 595 1/2 filter paper with pore size between 4–7 μm and found most of the microbubbles collapsed (Fig 3B). This porn size of the filter paper is in the similar range as the pulmonary capillaries which could mimic the crash of the microbubble in the lung. Mice bearing B16-F10 tumors were treated with saline, jetPEI-NC, jetPEI-siRNAc, microbubble-jetPEI-NC, microbubble-jetPEI-siRNAc, TfPEI-NC, and TfPEI-siRNAc complex in 7 groups with five mice in each group. For the microbubble coated jetPEI groups, all mice survived the procedure with no observable heart rate distress. The three siRNAs treatment groups showed significantly suppression of the growth of metastatic tumors comparing to the random sequence treated groups (Fig 3C–3E). Quantitative data are achieved by counting the numbers of the metastatic melanoma colonies in lung in 5 different classes: > 2 mm, 1.5–2 mm, 1–1.5 mm, 0.5–1.0 mm, < 0.5 mm. The number of metastatic colonies was summed by using different weight coefficient based on the square of the diameters since the height of the lung tissue between two glassed was the same. Statistical significance was analysed by Student's t-test. The data showed both Tf-PEI-siRNAs and Microbubble jet-PEI-siRNAs groups are better than jetPEI group. There is not significant different between Tf-PEI-siRNAs and Microbubble jet-PEI-siRNAs treatment groups (Fig 3F and Fig 3G). Moreover, the mice treated with random sequence also showed a tumor metastasis in the gastrointestinal tract and liver, all the groups treated with siRNAc had a remarkably inhibition of melanoma metastasis in gastrointestinal tract (Fig 3H–3K).

### Systemic administration of siRNA complex is not toxic to C57BL/6 mice

**Fig 4:**
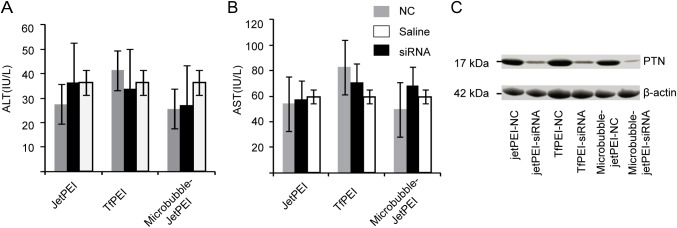
Monitoring the liver toxicity of mice in different treatment groups. Mice liver enzyme ALT (A) and AST (B) levels were detected at the end of study. There was no significant difference among the groups. The enzyme levels were in the same range as the group treated with normal saline. Data were shown as the mean ± SD. (C) Western blotting analysis of the PTN expression level in the lungs of mice in different treatment groups.

In the next step, we took resections of the lungs of mice with different treatments and homogenize them. The lung tissue lysate was then used for western blotting. Fig 4C confirmed that the siRNA reached lung and the expression level of PTN was reduced significantly. To monitor the safety of the systemic administration of siRNA-complex, the serum enzyme levels (ALT/AST) were evaluated. Equal volumes of serum samples were collected immediately at the time of killing. The ALT and AST values of all the mice injected with different siRNA–complexes or normal saline showed no significant changes (Fig 4A and Fig 4B) by Student's t-test. In addition, there was no elevation in serum enzyme levels during the treatment. The ALT and AST level were remained within the normal range for C57BL/6 mice when measured after the last time injection of siRNA-complex, indicating that repeated systemic administration of siRNA-complex did not cause damage to the liver. Taken together, these results suggest that these treatments with jetPEI-siRNA, Tf-PEI-siRNA, and microbubble-jetPEI-siRNA were well tolerated by the mice, providing a safe and specific inhibition of melanoma tumor growth.

## Discussion

RNAi has become a standard procedure in many research laboratories. It has potential for the treatment of various diseases. However, several studies reported about the off-target effects of the highly specific siRNA[29–31], which arises from the partial sequence complementarity of the siRNA used with non-targeted mRNA and competition with cellular micro RNA processes. High concentration of the individual or combination of siRNA may represent the main reason for the off-target effect. They may compete with the endogenous pool of miRNA for the binding with the RNA-induced silencing complex[32,33]. Thus a combination of low concentration of each siRNA could reduce the off-target phenomenon. In our study, we also observed higher silencing efficiency by combination of two siRNAs targeted to different locations of the same PTN mRNA. Consequently, a significant suppression of the tumor cell growth *in vitro* was also detected. PTN was reported to bind to RPTPβ/ζ and then indirectly activate the anaplastic lymphoma kinase (ALK). In PTN-stimulated cells, the RPTPβ/ζ could no longer dephosphorylate the sites that are autophosphorylated in ALK, resulting in autoactivation and tyrosine phosphorylation of ALK. The activation of ALK involved in the signaling pathway of proliferation, anti-apoptosis[7,34,35]. This is consistent with our data that the recombinantly expressed PTN stimulates the cell growth (Fig 1B). On the other hand, the reduced expression level of PTN by siRNA could result in less autoactivation of ALK and decreased proliferation rate (Fig 2C). In like manner, the antibody of the PTN receptor RPTPβ/ζ also inhibits the cell proliferation stimulated by PTN (Fig 1C).

For the systemic use of the siRNA to mediate gene silencing *in vivo*, an appropriate delivery system is required to stabilize the siRNA and enable the entry of siRNA into the cytosol of the cells. In this study, we compared the therapeutic effect of three different ways to deliver a mixture of 2'-deoxy modified siRNA in mice. All the siRNA treated groups showed a significant inhibition of the tumor growth in lung and also a remarkably inhibition of melanoma metastasis in gastrointestinal tract comparing to the random sequence treated group. As we expected, the cancer cell targeted delivery of siRNA complex by TfPEI suppressed more micrometastasis growth. Comparing to the previous studies[4,14], our experiments performed with the non-immune deficiency C57BL/6 mice to evaluate the therapeutic effects in a full immunity activity scenario. In addition, the silencing of PTN was also reported to inhibit the tumor angiogenesis *in vivo* by counting the blood vessels[4], indicating PTN is a promising target for the therapy of melanoma. We also propose an improvement of lung targeted delivery of jetPEI-siRNAc complex by coating it with microbubble. The microbubble is suitable because the melanoma metastases mainly located in the lung and the microbubble jetPEI-siRNAc complex after tail vein injection will go through the lung capillary bed first. Previous study on large microbubbles revealed that they coalesced in the capillary and subsequently to shrink to flow through or collapse[36]. Most microbubbles larger than 10 μm will be filtered by the pulmonary capillary[21] in consistent with our microscopic image of the microbubbles after passing through the grade 595 1/2 filter paper (Fig 3A and Fig 3B). In our study, all the mice are well tolerated with the size of the microbubbles showing no observable heart distress. The diameter of the microbubbles as large as 19μm was revealed to be safe from a recent *in vivo* imaging of microbubble study[37]. The combination of therapeutic jetPEI-siRNA complex with large size microbubbles revealed an efficient and lung targeted gene delivery system. Significant less melanoma metastases was found in the lung comparing to using jetPEI-siRNA alone, indicating a potential application for aiding systemic delivery of siRNA *in vivo* in future.

## Supporting information

S1 Checklist ARRIVE guidelines checklist. (DOCX)
