# Supplementary material for: Therapeutic silence of pleiotrophin by targeted delivery of siRNA and its effect on the inhibition of tumor growth and metastasis
Source: PLoS One. 2017 May 31;12(5):e0177964. doi: 10.1371/journal.pone.0177964 (PMC5451024; doi:10.1371/journal.pone.0177964)

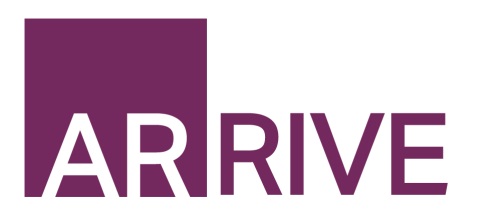


The ARRIVE Guidelines Checklist

Animal Research: Reporting In Vivo Experiments

Carol Kilkenny^1^, William J Browne^2^, Innes C Cuthill^3^, Michael Emerson^4^ and Douglas G Altman^5^

*^1^The National Centre for the Replacement, Refinement and Reduction of Animals in Research, London, UK, ^2^School of Veterinary Science, University of Bristol, Bristol, UK, ^3^School of Biological Sciences, University of Bristol, Bristol, UK, ^4^National Heart and Lung Institute, Imperial College London, UK, ^5^Centre for Statistics in Medicine, University of Oxford, Oxford, UK.*

|  | | ITEM | RECOMMENDATION | Section/ Paragraph |
| --- | --- | --- | --- | --- |
| 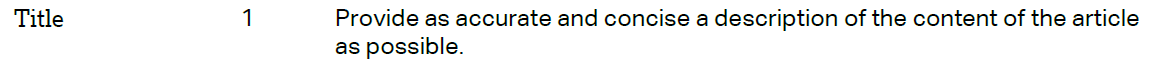 | | | Title |  |
| 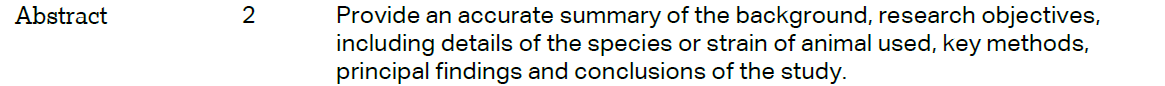 | | | Abstract |  |
| INTRODUCTION | | |  |  |
| 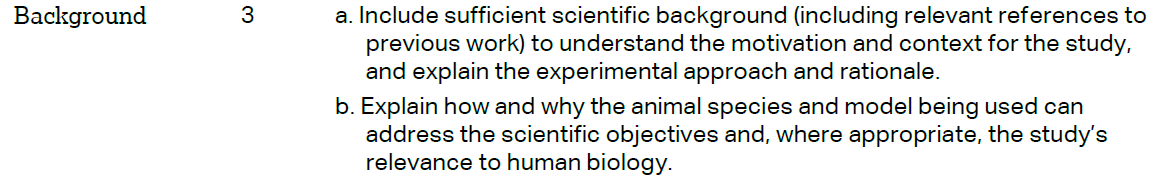 | | | Paragraph 1-2  Paragraph 2-3 |  |
| 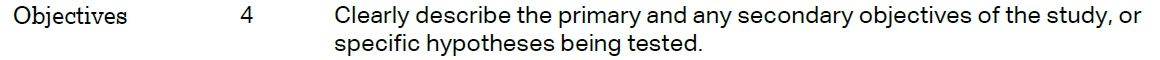 | | | Paragraph 2-3 |  |
| METHODS | | |  |  |
| 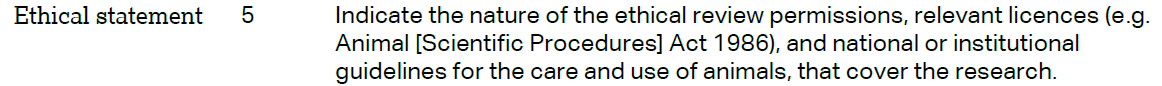 | | | Method  Paragraph 5 |  |
| 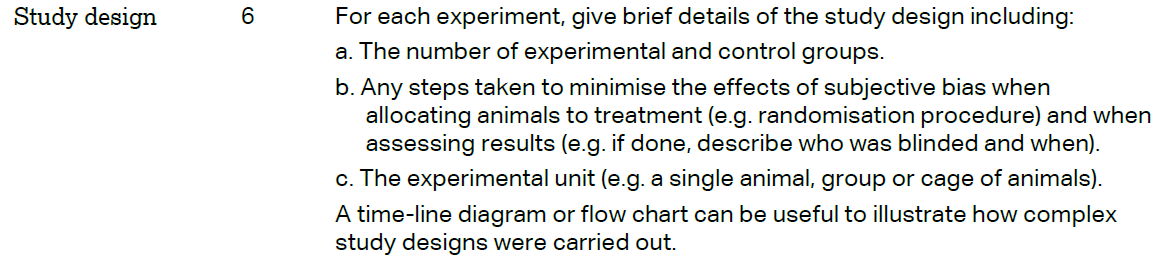 | | | Method  Paragraph 5  Method  Paragraph 6 |  |
| 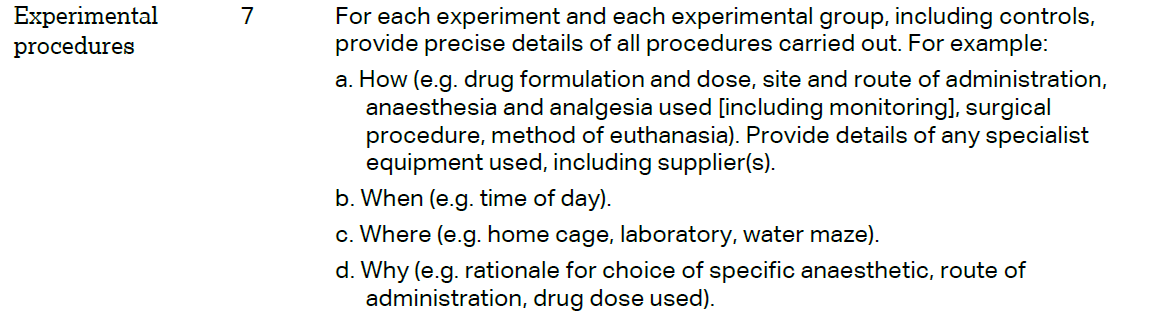 | | | Method  Paragraph 4  Method  Paragraph 5 |  |
| 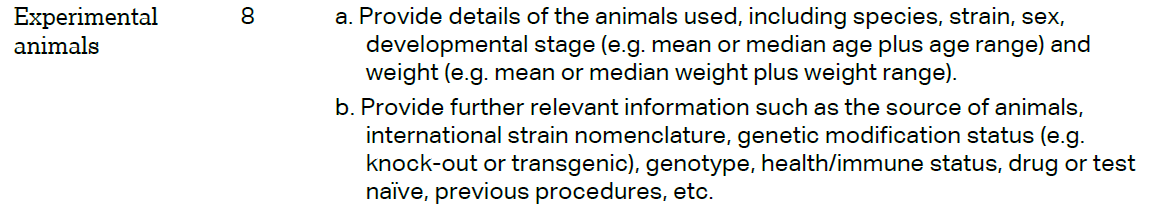 | | | Method  Paragraph 5 |  |

The ARRIVE guidelines. Originally published in *PLoS Biology*, June 2010^1^

| 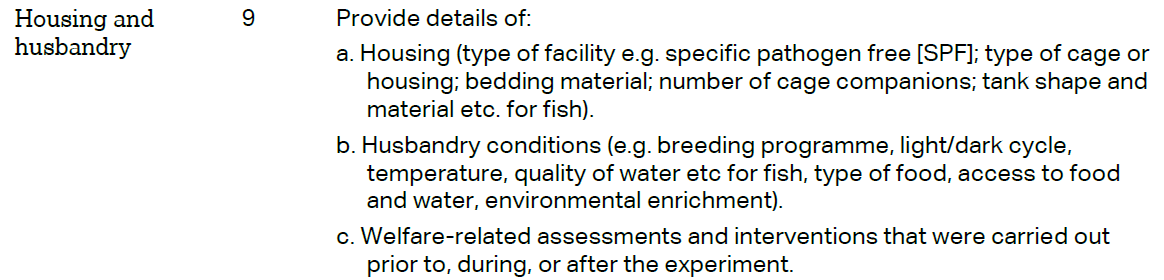 | Method  Paragraph 5 | |
| --- | --- | --- |
| 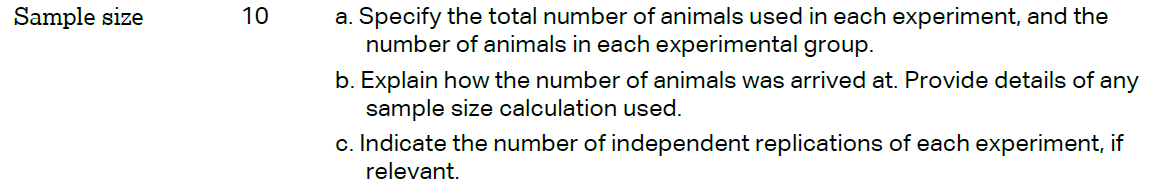 | Method  Paragraph 5  Method  Paragraph 6  Fig 3 | |
| 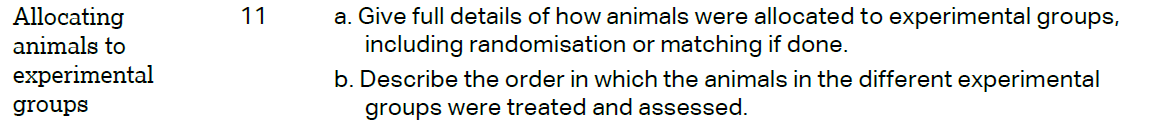 | Method  Paragraph 5 | |
| 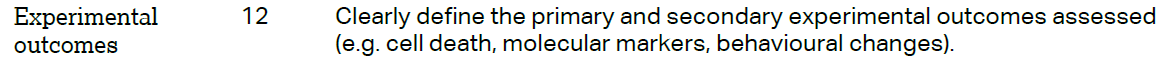 | Result  Paragraph 4 | |
| 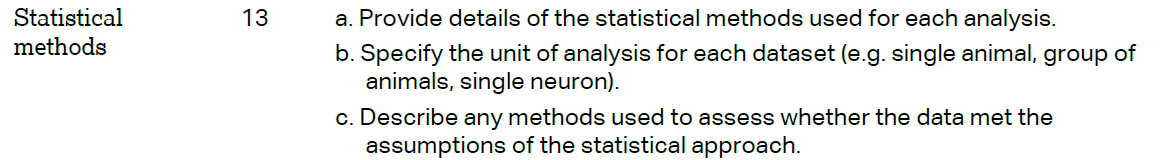 | Method  Paragraph 6  Fig 3 | |
| RESULTS |  | |
| 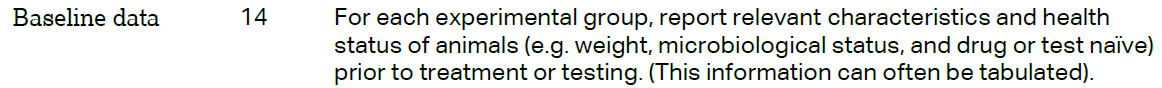 | Fig 3  Fig 4 | |
| 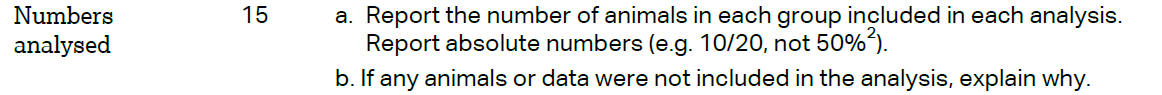 | Method  Paragraph 5  Fig 3  Method  Paragraph 6 | |
| 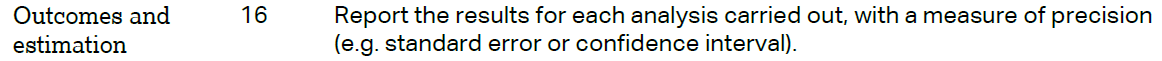 | Method  Paragraph 6  Fig 3 Fig 4 | |
| 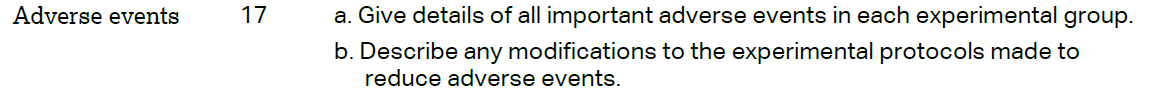 |  | |
| DISCUSSION |  | |
| 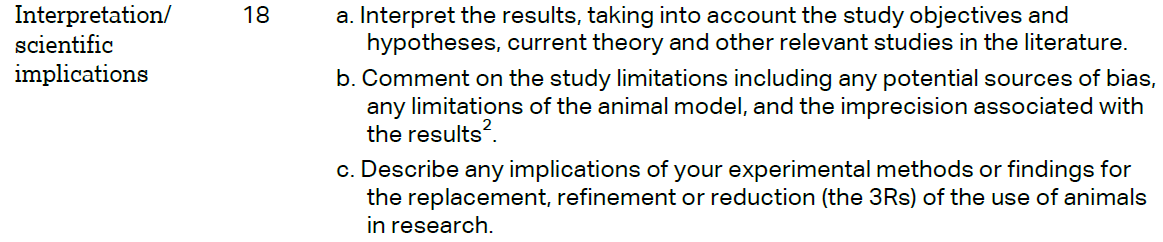 | Method  Paragraph 6  Fig 3 Fig 4 | |
| 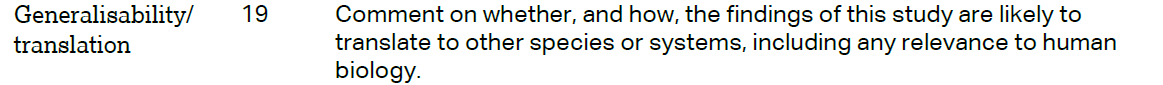 | Discussion  Paragraph 2 | |
| 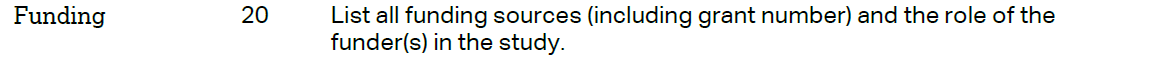 | |  |


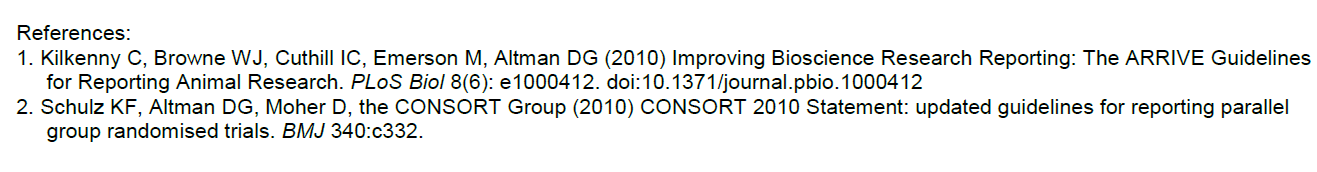

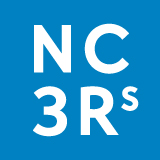

Supplement: S1 Checklist — (DOCX) [file pone.0177964.s001.docx]
